# Assessment of the Nasopalatine Canal in Patients Requiring Dental Implants in the Maxillary Anterior Region Using Cone Beam Computed Tomography

**DOI:** 10.7759/cureus.50643

**Published:** 2023-12-16

**Authors:** Sandeep K Bains, Archana Bhatia, Surenderpal S Sodhi, Ashwarya Sharma

**Affiliations:** 1 Department of Oral Medicine and Radiology, Surendera Dental College and Research Institute, Sri Ganganagar, IND; 2 Department of Periodontology and Implantology, Surendera Dental College and Research Institute, Sri Ganganagar, IND; 3 Department of Oral and Maxillofacial Surgery, Dasmesh Institute of Research & Dental Sciences, Faridkot, IND; 4 Department of Oral Medicine and Radiology, Guru Nanak Dev Dental College & Hospital, Sunam, IND

**Keywords:** anatomical assessment, maxillary anterior region, nasopalatine canal, dental implant, cone beam computed tomography

## Abstract

Aim: To assess the nasopalatine canal in patients requiring dental implants in the maxillary anterior region using cone beam computed tomography (CBCT).

Methodology: About 56 patients requiring dental implants in the maxillary anterior region of either gender reporting to the daily Outpatient Department, Department of Oral Medicine and Radiology aged between 18 and 60 years were selected. They were subjected to a CBCT scan using Newtom Giano CBCT machine (NewTom, Imola, Italy). Newtom New Technology software and a slice thickness of 0.3 mm were used. For assessment of the implant site at the maxillary anterior region, alveolar bone width in the anterior region of the canal at the upper, middle, and lower third, incisive foramen diameter (IFD), nasopalatine canal length (NPCL), canal diameter in the floor of the nasal fossa and nasopalatine canal morphology was determined.

Results: Out of 56 patients, males comprised 30 and females 26. The mean bone height in 11 regions was 15.6±3.9, in 12, 16.2±2.7 mm, in 13 was 15.5±2.4 mm, in 21 was 13.7±4.2 mm, in 22 was 14.7±3.6 mm, in 23 was 16.7±1.5 mm. A non-significant difference was observed when comparing the bone height at different implant sites (p>0.05). The mean bone width at 3 mm and 6 mm from an alveolar crest in 11 regions was 4.3 mm and 5.3 mm, in 12 was 5.7 mm and 6.2 mm, in 13 was 3.7 mm and 4.6 mm, in 21 regions was 4.1 mm and 5.5 mm, in 22 regions was 4.6 mm and 5.7 mm and in 23 regions was 4.0 mm and 4.9 mm, respectively. A significant difference was observed when comparing the bone width at 3 mm and 6 mm at different implant sites (p<0.05). Nasopalatine canal type was A in 42 (75%), B in 13 (23.2%), and C in 1 (1.8%) patient. The mean alveolar bone width in the anterior region of the canal at the upper third was 10.1 mm, in the middle third was 7.4 mm, and in the lower third was 5.2 mm. The mean IFD was 4.6 mm, the NPCL was 13.5 mm, and the canal diameter on the floor of the nasal fossa was 3.8 mm. A significant difference was observed in comparing nasopalatine canal type (p<0.05).

Conclusion: For the clinician to assess implant placement in the maxillary esthetic zone, CBCT imaging of the nasopalatine canal is crucial. It is possible to prevent intraoperative and postoperative complications such as hemorrhage, sensory impairment, osseointegration failure, and nasopalatine duct cyst formation.

## Introduction

Dental rehabilitation programs for individuals with missing teeth can be managed with dental implants. The clinician chooses the optimum implant placement sites by considering anatomical and prosthetic aspects. Wherever there is the greatest probability of success, dental implants should be inserted in that site [1]. Implant treatment depends on efficient planning, which includes assessment of height, width, and morphology and identification and location of anatomical landmarks. Recommendations for successful results ideally require at least 1 mm of bone surrounding each implant [2]. Careful evaluation of anatomical landmarks such as nasopalatine canal, nasal fossae, maxillary sinus, zygomatic bone, pterygoid process, inferior alveolar nerve canal, mental foramen, incisive canal, and lingual foramen helps in treatment planning [3]. An essential anatomical landmark, the nasopalatine canal plays a significant role in implant implantation. It is in the middle of the maxilla, between and slightly posterior to the central maxillary incisors. It has an average length of 10 mm and an average width of up to 6 mm at the incisive fossa. The Stenson foramen allows the nasopalatine canal to enter the nasal cavity, and the incisive foramen extends into the oral cavity. With tiny salivary glands, fat, and fibrous connective tissue, the nasopalatine canal also houses the nasopalatine nerve, the terminal branch of the descending nasopalatine artery.

An incorrect assessment of the canal's placement could result in injury and perforation, which would traumatize the nerves and blood vessels and cause paresthesia in the premaxilla. Therefore, the anatomy and size of the nasopalatine canal should be carefully assessed before placing dental implants. Nasopalatine canal has been classified according to morphology into type A, which is a cylindrical canal without any branches, type B, which is a canal with a branch in the upper part; and type C, with a branch in the middle part [4]. The craniofacial complex can now be seen in three dimensions using cone beam computed tomography (CBCT) equipment. Similar to multi-slice conventional computed tomography (CT), CBCT generates images and volumetric reconstructions of craniofacial features; however, it does so with shorter acquisition times, lower effective radiation doses, and a lighter financial load than CT [5]. With the advent of CBCT technology, it is now possible to evaluate dental and craniofacial anatomy in three-dimensional (3D) without being constrained by the constraints of traditional two-dimensional (2D) imaging [6]. By performing a multidimensional, presurgical anatomy assessment using CBCT, it is possible to lower the risk of implant placement errors, which might have unfavorable consequences like invasions of nearby structures and perforations of cortical borders [7]. The present study was conducted to assess the nasopalatine canal in patients requiring dental implants in a maxillary anterior region using CBCT.

## Materials and methods

The prospective study was conducted at Dasmesh Institute of Research and Dental Sciences, Faridkot, India on 56 patients (males - 30, females - 26) requiring dental implants in the maxillary anterior region of either gender reporting to the daily Outpatient Department, Department of Oral Medicine and Radiology, aged between 18 and 60 years. The duration of the study was one year. All the patients were briefed about the study, and written informed consent in the local language was obtained. The Institute’s Ethics Committee issued approval (DIRDS/IEC/2019/84) to carry out the study.

Inclusion criteria were patients aged 18-80 years requiring dental implants in the maxillary anterior region and giving consent. Exclusion criteria were patients with jaw fractures, the presence of root pieces, nasopalatine pathologies such as nasopalatine duct cysts, patients undergoing orthodontic treatment, pregnant women, and poor-quality images.

Data such as name, age, gender, etc., was recorded. A careful oral examination was done. Patients were subjected to CBCT scan using Newtom Giano CBCT machine (NewTom, Imola, Italy) operating at 90 kVp and 1-10mA with a field of view ranging from 5X5 cm to 11X8 cm, exposure time 3.6 seconds, and voxel size of 0.3 mm X 0.3 mm X 0.3 mm. Newtom New Technology (NNT) software and a slice thickness of 0.3 mm were used. All three planes, such as the coronal, sagittal, and axial, were obtained. For assessment of implant site at the maxillary anterior region, bone height, width, alveolar bone width in the anterior region of the canal at upper, middle, and lower third, incisive foramen diameter (IFD), nasopalatine canal length (NPCL), canal diameter in the floor of the nasal fossa (CDNF) and nasopalatine canal morphology was determined. Data were compiled and entered in Excel 2010 (Microsoft, Redmond, United States). Data were subjected to analysis of variance (ANOVA one-way), and t-test and analyzed using the IBM SPSS Statistics for Windows, Version 19 (Released 2010; IBM Corp., Armonk, New York, United States). Descriptive statistics (frequency, percentage, mean) were done. p-value < 0.05 was considered significant.

## Results

**Figure 1:**
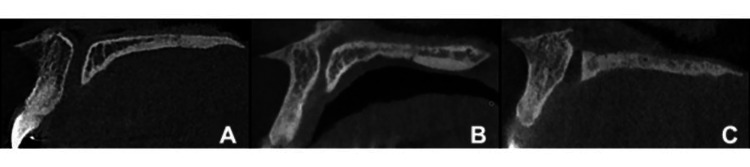
CBCT-images showing the three shapes of the nasopalatine canal A: Cylindrical shape (type A); B: A canal with a branch in the upper part (type B); C: A canal with a branch in the middle part (type C) CBCT: Cone beam computed tomography

Based on the labial and palatal walls of the nasopalatine canal, CBCT scans display the three forms of the canal on sagittal planes. The nasopalatine canal's parallel labial and palatal walls produce a cylindrical shape (type A), a canal with a branch in the upper part (type B), and a canal with a branch in the middle part (type C) (Figure 1). 

**Figure 2:**
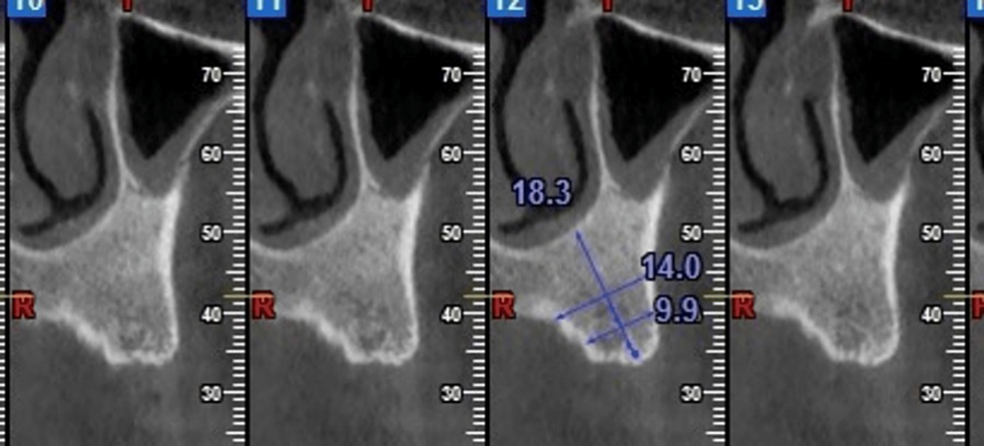
Measurement of bone height and bone width

The following figure illustrates the CBCT measurements of bone height and bone width at different implant sites. These measurements are crucial for assessing the suitability of implant placement in the maxillary anterior region (Figure 2).

**Figure 3:**
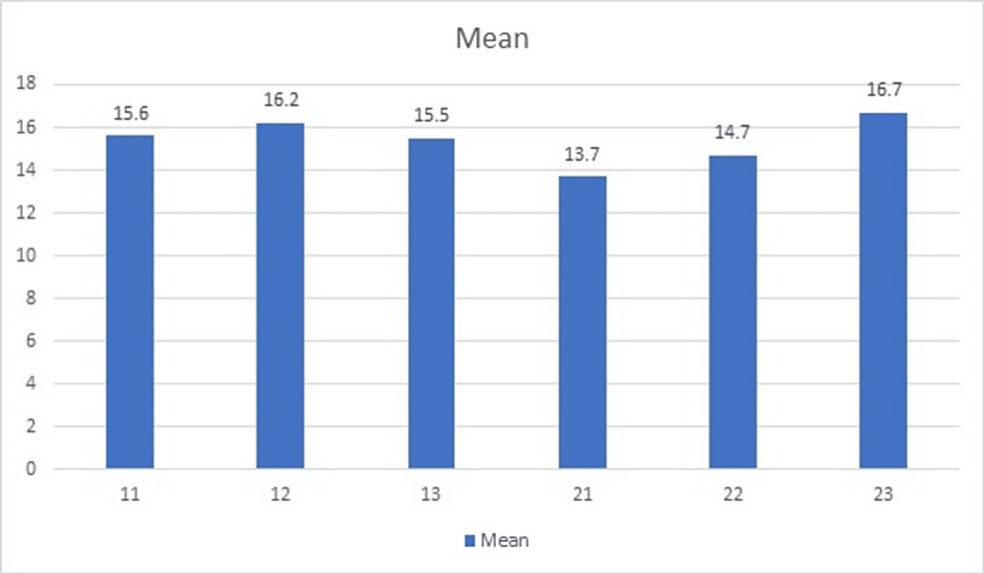
Assessment of bone height

Out of 56 patients, males comprised 30 and females 26. The mean bone height in 11 regions was 15.6±3.9, in 12 was 16.2±2.7 mm, in 13 was 15.5±2.4 mm, in 21 was 13.7±4.2 mm, in 22 was 14.7±3.6 mm, in 23 was 16.7±1.5 mm. A non-significant difference was observed when comparing the bone height at different implant sites (p>0.05) (Table 1, Figure 3).

**Table 1:** Assessment of bone height

Tooth region	Mean	SD	p-value
11	15.6	3.9	0.71
12	16.2	2.7	
13	15.5	2.4	
21	13.7	4.2	
22	14.7	3.6	
23	16.7	1.5	

The mean bone width at 3 mm and 6 mm from an alveolar crest in 11 regions was 4.3 mm and 5.3 mm, in 12 was 5.7 mm and 6.2 mm, in 13 was 3.7 mm and 4.6 mm, in 21 regions was 4.1 mm and 5.5 mm, in 22 regions was 4.6 mm and 5.7 mm and in 23 regions was 4.0 mm and 4.9 mm, respectively. A significant difference was observed when comparing the bone width at 3 mm and 6 mm at different implant sites (p<0.05) (Table 2). 

**Table 2:** Assessment of bone width

Implant sites (tooth region)	3 mm		6 mm	
	Mean	SD	Mean	SD
11	4.3	1.3	5.3	1.0
12	5.7	1.7	6.2	1.4
13	3.7	1.5	4.6	1.4
21	4.1	1.3	5.5	1.1
22	4.6	1.4	5.7	1.4
23	4.0	0.8	4.9	0.4
f-value	7.4		12.1	
p-value	0.001		0.001	

Nasopalatine canal type was A in 42 (75%), B in 13 (23.2%), and C in 1 (1.8%) patient. The mean alveolar bone width in the anterior region of the canal at the upper third was 10.1 mm, in the middle third was 7.4 mm, and in the lower third was 5.2 mm. The mean IFD was 4.6 mm, NPCL was 13.5 mm, and CDNF was 3.8 mm. A significant difference was observed in comparing nasopalatine canal type (p<0.05) (Table 3).

**Table 3:** Assessment of nasopalatine canal

Parameters	Variables	Number/mean
Nasopalatine canal type	A	42 (75%)
	B	13 (23.2%)
	C	1 (1.8%)
	p-value	0.001
Alveolar bone width in the anterior region of the canal (mm)	Upper third	10.1±2.3
	Middle third	7.4±1.8
	Lower third	5.2±0.7
Incisive foramen diameter (IFD) (mean±SD) (mm)		4.6±0.6
Nasopalatine canal length (NPCL) (mean±SD) (mm)		13.5±1.6
Canal diameter in the floor of the nasal fossa (CDNF) (mean±SD) (mm)		3.8±0.6

## Discussion

A thorough patient evaluation is necessary for effective treatment planning and dental implant placement. The precision of treatment planning with earlier traditional radiography techniques, such as intraoral, cephalometric, and panoramic pictures, was compromised by imaging distortion and superimposition [8]. The recommendation to employ tomographic techniques to examine possible implant locations resulted from advancements in sectional imaging techniques. The data set can be seen in the multiplanar reconstruction perspective using CBCT NNT software. Axial, coronal, and sagittal planes can be used to see the images. This enables the design of dental implants at a suggested location that may be evaluated from various angles [9]. The implant gallery contains a variety of implants, allowing for selecting a particular dental implant as needed. Setting the default values for the surface smooth, edge enhancer, and model quality is possible with a volume 3D reconstruction. To analyze both bone and soft tissues, it offers numerous viewpoints. A moderate tissue contrast is provided by CBCT [10].

According to the American Academy of Oral and Maxillofacial Radiology (AAOMR), the most effective choice is CBCT. For preoperative cross-sectional imaging of possible implant locations, the AAOMR advised that CBCT be considered the imaging modality of choice [11]. The present study was conducted to assess the nasopalatine canal in patients requiring dental implants in the maxillary anterior region using CBCT.

In our study, out of 56 patients, males comprised 30 and females 26. We observed that the mean bone height in 11 regions was 15.6±3.9, in 12 was 16.2±2.7 mm, in 13 was 15.5±2.4 mm, in 21 was 13.7±4.2 mm, in 22 was 14.7±3.6 mm, in 23 was 16.7±1.5 mm (Table 1). Sathvik et al. [12] used CBCT to evaluate the nasopalatine duct's position and size in 50 dentulous and edentulous instances, with patients' ages ranging from 23 to 80. Of these, 15 were men and 9 were women. Males had a mean canal length of 14.69 mm, while females had a mean canal length of 12.74 mm from the anterior wall of the foramen to the anterior nasal spine.

We found that the mean bone width at 3 mm and 6 mm from an alveolar crest in 11 regions was 4.3 mm and 5.3 mm, in 12 was 5.7 mm and 6.2 mm, in 13 was 3.7 mm and 4.6 mm, in 21 regions was 4.1 mm and 5.5 mm, in 22 regions was 4.6 mm and 5.7 mm and in 23 regions was 4.0 mm and 4.9 mm, respectively (Table 2). Chatzipetros et al. [13], in their study, 124 CBCT scans in total (67 female patients and 57 male patients) were included and assessed retrospectively. The mean values for the nasopalatine canal and neighboring buccal osseous plate dimensions were noticeably greater in males than in females. Nasopalatine canal types had a significant impact on the length of the nasopalatine canal. Age significantly affected the diameter of the incisive foramen, with the mean values generally increasing with age. CBCT imaging of this anatomical structure contributes significantly to its full assessment.

We observed that nasopalatine canal type was A in 42 (75%), B in 13 (23.2%), and C in 1 (1.8%) patient. The mean alveolar bone width in the anterior canal region at the upper third was 10.1 mm, in the middle third was 7.4 mm, and in the lower third was 5.2 mm. The mean IFD was 4.6 mm, NPCL was 13.5 mm, and CDNF was 3.8 mm (Table 3). Panjnoush et al. [14] observed that a total of 199 individuals (66.3%) had type A canals, 69 (23%) had type B canals, and 32 (10.7%) had type C canals. On the sagittal slice, the diameter of the incisive foramen was 4.7±1.11 mm. Alveolar bone width measured 12.3±1.7 mm in the top third, 10.7±1.7 mm in the middle third, and 9.8±1.4 mm in the lower third of the canal's anterior portion. On the coronal sections, the nasal floor canal's diameter was 5.1 mm, the incisive foramen's diameter was 4.6 mm, and the canal length was 14.1 mm by 3.0 mm.

CBCT has opened up new diagnostic possibilities for dental imaging by extending the limits of imaging from 2D to 3D [15]. Since CBCT provides a relatively low radiation dose and good picture quality, it has become the preferred volumetric 3D imaging method among general dentistry practitioners and specialists. However, poor soft tissue contrast, high cost, and metal artifacts are its drawbacks.

This study may be useful in providing information regarding nasopalatine canal morphology and its role in determining the success of dental implants in the maxillary esthetic region. This study also highlighted the importance of CBCT in dental implant planning. 

The limitation of the study is the small sample size, and the effect of age on the nasopalatine canal was not studied.

## Conclusions

The authors found that for the clinician to assess implant placement in the maxillary esthetic zone, CBCT imaging of the nasopalatine canal is crucial. It is possible to prevent intraoperative and postoperative complications such as hemorrhage, sensory impairment, osseointegration failure, and nasopalatine duct cyst formation.

## References

[1] Rajiv S (2013). Dental implants: a review. RRJDS.

[2] Geckili O, Bilhan H, Geckili E, Cilingir A, Mumcu E, Bural C (2014). Evaluation of possible prognostic factors for the success, survival, and failure of dental implants. Implant Dent.

[3] Gil-Marques B, Sanchis-Gimeno JA, Brizuela-Velasco A, Perez-Bermejo M, Larrazábal-Morón C (2020). Differences in the shape and direction-course of the nasopalatine canal among dentate, partially edentulous and completely edentulous subjects. Anat Sci Int.

[4] Nasseh I, Aoun G, Sokhn S (2017). Assessment of the nasopalatine canal: an anatomical study. Acta Inform Med.

[5] Thakur AR, Burde K, Guttal K, Naikmasur VG (2013). Anatomy and morphology of the nasopalatine canal using cone-beam computed tomography. Imaging Sci Dent.

[6] Chau AC, Fung K (2009). Comparison of radiation dose for implant imaging using conventional spiral tomography, computed tomography, and cone-beam computed tomography. Oral Surg Oral Med Oral Pathol Oral Radiol Endod.

[7] Bornstein MM, Balsiger R, Sendi P, von Arx T (2011). Morphology of the nasopalatine canal and dental implant surgery: a radiographic analysis of 100 consecutive patients using limited cone-beam computed tomography. Clin Oral Implants Res.

[8] Mraiwa N, Jacobs R, Van Cleynenbreugel J (2004). The nasopalatine canal revisited using 2D and 3D CT imaging. Dentomaxillofac Radiol.

[9] Al-Ghurabi ZH, Al-Bahrani ZM (2020). Radiographic assessment of nasopalatine canal using cone beam computed tomography. J Craniofac Surg.

[10] Gönül Y, Bucak A, Atalay Y (2016). MDCT evaluation of nasopalatine canal morphometry and variations: an analysis of 100 patients. Diagn Interv Imaging.

[11] Tyndall DA, Price JB, Tetradis S, Ganz SD, Hildebolt C, Scarfe WC (2012). Position statement of the American Academy of Oral and Maxillofacial Radiology on selection criteria for the use of radiology in dental implantology with emphasis on cone beam computed tomography. Oral Surg Oral Med Oral Pathol Oral Radiol.

[12] Sathvik N, Nessapan T, Dhanraj M (2018). Assessment of position and size of nasopalatine duct in dentulous and edentulous patients using cone-beam computed tomography: a retrospective study. Drug Invention Today.

[13] Chatzipetros E, Tsiklakis K, Donta C, Damaskos S, Angelopoulos C (2023). Morphological assessment of nasopalatine canal using cone beam computed tomography: a retrospective study of 124 consecutive patients. Diagnostics (Basel).

[14] Panjnoush M, Norouzi H, Kheirandish Y, Shamshiri AR, Mofidi N (2016). Evaluation of morphology and anatomical measurement of nasopalatine canal using cone beam computed tomography. J Dent (Tehran).

[15] Choudhary A, Kesarwani P, Verma S (2021). Comparative study of implant site assessment using CBCT, tomography and panoramic radiography. Journal of Indian Academy of Oral Medicine and Radiology.

